# Effect of SGLT2 Inhibitors on the Efficacy of First-Time Pulmonary Vein Isolation and Clinical Course of Patients with Heart Failure with Preserved Ejection Fraction and Atrial Fibrillation

**DOI:** 10.3390/jcdd13040160

**Published:** 2026-04-06

**Authors:** Magdalena Balwierz-Podgórna, Bartosz Gruchlik, Katarzyna Mizia-Stec, Adriana Filak, Anna Hejmej, Piotr Paździora, Mikołaj Gołda, Aleksandra Spirkowicz, Karina Dzięcioł, Maciej Wybraniec

**Affiliations:** 1 First Department of Cardiology, School of Medicine in Katowice, Medical University of Silesia, 40-635 Katowice, Poland; 2 School of Medicine in Katowice, Medical University of Silesia, Upper-Silesian Medical Center, 40-635 Katowice, Poland

**Keywords:** SGLT2 inhibitors, PVI efficacy, heart failure with preserved ejection fraction (HFpEF)

## Abstract

**Background/Objectives**: SGLT2 inhibitors (SGLT2i) became a cornerstone of heart failure with preserved ejection fraction (HFpEF) pharmacotherapy in the recent years However, their actual influence on pulmonary veins isolation (PVI) efficacy in this population remains unclear. The aim of the study was to evaluate an impact of SGLT2i on one-year first-time PVI efficacy and clinical course of patients with HFpEF and atrial fibrillation (AF). **Methods**: This is a single-center retrospective study including 105 HFpEF and AF individuals, who underwent the first-time PVI (51 (48.6%) males; mean age at PVI: 65.2 ± 9.5 years). 53 patients treated with SGLT2i (hospitalized for PVI since 2023) and 52 patients without such a treatment (2020-mid-2023) were assessed according to the clinical presentation and hard endpoints. The primary endpoint was arrhythmia recurrence rate. The secondary endpoint was a composite of major adverse cardiovascular and cerebrovascular events (MACCE). **Results**: SGLT2i therapy was associated with greater symptom reduction after PVI (90.6% vs. 62.7%; *p* < 0.001). There was a statistical trend toward reduced all-cause mortality in SGLT2i (0% vs. 5.8%; *p* = 0.076). Although overall AF recurrence rates were similar between subgroups, Kaplan–Meier analysis showed a non-significant trend toward lower recurrence in the SGLT2i group (*p* = 0.096). The analysis did not reveal significant differences in terms of cardiovascular hospitalizations, stroke/transient ischemic attack (TIA) and MACCE incidence between the subgroups. Non-vitamin K antagonist oral anticoagulants (NOACs) administration was associated with a lower risk of AF recurrence (OR 0.27; 95% CI 0.096 to 0.77; *p* = 0.014). MACCE occurrence was predicted by higher CHA_2_DS_2_-VA (Congestive heart failure, Hypertension, Age ≥ 75, Diabetes, Stroke, Vascular disease, Age 65–74) (OR 5.63; 95% CI 1.57–20.12; *p* = 0.008), lower left ventricular ejection fraction (LVEF) (OR 0.74; 95% CI 0.57–0.99; *p* = 0.028) and (vitamin K antagonists) VKA use (OR 97.44; 95% CI 3.2–2962.57; *p* = 0.009). **Conclusions**: SGLT2i pharmacotherapy in the study population was linked to higher efficacy in symptom reduction, with a probability of AF recurrence and all-cause mortality reduction, which may suggest a potential beneficial role of SGLT2i in this cohort.

## 1. Introduction

Sodium-glucose co-transporter 2 (SGLT2) inhibitors, primarily indicated for the treatment of type 2 diabetes mellitus (T2DM), have recently become a fundamental component of heart failure (HF) treatment, including heart failure with preserved ejection fraction (HFpEF). This pharmacological class has demonstrated substantial cardiovascular benefits, including reduction in HF hospitalization rates, symptom severity and improvement in quality of life [1,2,3,4]. These effects are attributed to the combination of hemodynamic modulation, improved mitochondrial function, anti-inflammatory and antifibrotic properties, as well as reduction in left atrial pressure and volume overload [5,6]. HFpEF represents nearly 50% of all HF cases and is frequently associated with atrial fibrillation (AF) [7,8]. The coexistence of HFpEF and AF is particularly challenging, as each condition promotes the progression of the other [9]. HFpEF is related to left atrial dilation and elevated filling pressures, whereas AF exacerbates diastolic dysfunction and impairs the atrial contribution to ventricular filling [10]. This bidirectional interaction results in a complex clinical phenotype characterized by poor exercise tolerance, elevated hospitalization rates and increased mortality [11,12]. Pulmonary vein isolation (PVI) remains the primary interventional strategy for achieving rhythm control in patients with symptomatic or drug-refractory AF [13,14]. Despite its proven efficacy, the efficacy of PVI in HFpEF individuals may be limited by structural atrial remodeling, fibrosis, and elevated atrial pressures, which contribute to higher recurrence rates [15,16]. Recent data suggest that SGLT2 inhibitors may exert protective effects on the myocardium by reducing oxidative stress, inflammation and fibrosis, thereby potentially improving the atrial substrate for ablation. Additionally, by improving diastolic function and decreasing left atrial pressure, SGLT2 inhibitors may enhance the procedural efficacy of PVI and promote durable rhythm stability [17].

## 2. Materials and Methods

### 2.1. Study Population

**Figure 1:**
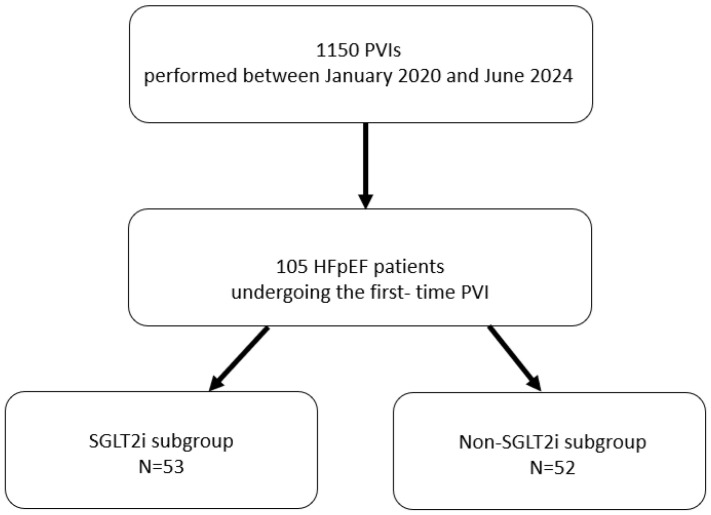
Flow-chart.

This was a retrospective, observational single center study conducted at the 1st Department of Cardiology of the Upper Silesian Medical Center in Katowice, Poland. During the observation period 1150 PVI procedures were performed. Out of this population 105 patients diagnosed with HFpEF and AF, who underwent the first-time PVI were identified. HFpEF diagnosis was made based on the ESC guidelines, which included: the presence of symptoms and/or signs of HF, LVEF ≥ 50% assessed by echocardiography and objective evidence of cardiac structural and/or functional abnormalities consistent with left ventricle (LV) diastolic dysfunction or elevated LV filling pressures, including increased natriuretic peptides levels. If necessary, the H2FPEF score with 6 points cut-off was used [18]. The study group was divided according to the pharmacological treatment at discharge: those receiving SGLT2 inhibitors (dapagliflozin or empagliflozin in dosages of 10 mg/day) according to the European Society of Cardiology guidelines [1] (patients hospitalized for PVI since 2023), and those without such a pharmacotherapy on admission and during the follow-up period (population hospitalized before 2023) (Figure 1. Flow chart). Pharmacotherapy with SGLT2 inhibitors was initiated from six months to one year before ablation and continued for at least one year after the procedure as a part of the follow-up protocol. The exclusion criteria were as follows: reduced left ventricular ejection fraction (LVEF) below 50%, absence of a confirmed diagnosis of HFpEF, undergoing repeat PVI, an arrhythmia of etiology other than AF, advanced chronic kidney disease (stage G4–G5), advanced-stage liver disease (Child–Pugh C), active infection or malignancy, autoimmune diseases or collagen vascular disorders with cardiac involvement, and inability to collect follow-up data one year after PVI. The study was conducted in accordance with the ethical standards outlined in the Declaration of Helsinki.

### 2.2. Data Collection

The study period spanned from January 2020 to June 2024. Data was obtained from the hospital’s electronic medical database. Assessments included evaluation of both quantitative and qualitative variables at baseline, as well as outcomes following PVI. Baseline variables involved: sex, age, body mass index (BMI), CHA_2_DS_2_-VA score, European Heart Rhythm Association (EHRA) classification, New York Heart Association (NYHA) functional class, type of AF (paroxysmal or non-paroxysmal), type of ablation, presence of comorbidities, transthoracic echocardiography (TTE) parameters and medication use (especially antiarrhythmic drugs (AADs) before and after PVI, and the type of anticoagulants).

### 2.3. PVI Characterization and AAD Treatment

The whole cohort underwent the first-time PVI. Each procedure was preceded by electrophysiologic study (EPS) and left atrial 3D mapping, performed using the CARTO 3 electroanatomical system (Biosense Webster, Irvine, CA, USA). Electrical isolation of pulmonary vein ostia was performed using either balloon cryoablation or radiofrequency (RF) ablation techniques. During cryoablation, Arctic Front Advance catheters (Medtronic, Minneapolis, MN, USA) were used. In the RF group, the following catheters were utilized: QDOT Micro (Biosense Webster, Irvine, CA, USA), TactiCath (Abbott, Santa Clara, CA, USA), TactiFlex (Abbott, Santa Clara, CA, USA) and SmartTouch Thermocool (Biosense Webster, Irvine, CA, USA). Antiarrhythmic drugs were prescribed for the blanking period of 3 months after PVI. The observation period extended from January 2020 to June 2024, and nearly all patients underwent the procedure in accordance with the indications outlined in the 2020 guidelines. To minimize further bias arising from potential differences in treatment protocols, the indications for the procedure and ablation techniques that might have influenced the results, patients from earlier years were intentionally excluded.

### 2.4. Follow-Up and Outcomes

The whole study group was assessed 12 months after PVI in an outpatient clinic. In case of symptomatic arrhythmia recurrence, before the clinic appointment, the patients were consulted at the emergency department. AF recurrence was defined as AF documented in single 12-lead electrocardiogram (ECG) (ECG GREY v.07.305SYS, ASPEL S.A., Zabierzów, Poland), ECG Holter monitoring (AsPEKT 712 v.301, ASPEL S.A., Zabierzów, Poland) after a blanking period of 3 months post procedure. The 48 h ECG Holter was performed at 12 months post procedure. Additionally, patients who experienced symptoms suggestive of arrhythmia recurrence before the one-year follow-up, underwent the 12-lead ECG or Holter monitoring, if necessary to document arrhythmia recurrence. This approach allowed detection of both asymptomatic and symptomatic atrial arrhythmias. Subsequent outcomes were evaluated and referred to their occurrence over the 12 months following PVI. The primary endpoint was the incidence of arrhythmia recurrence. The secondary endpoint encompassed major adverse cardiovascular and cerebrovascular events (MACCE), including all-cause mortality, cardiovascular-related hospitalizations, ischemic stroke or transient ischemic attack (TIA), other cardiovascular events, defined as acute coronary syndromes, and severe bleeding events. Severe bleeding was defined as fatal or symptomatic bleeding in a critical organ or area, or hemoglobin drop of at least 2 g/dL according to the International Society on Thrombosis and Haemostasis definition [19]. Symptom burden was evaluated based on structured clinical evaluation and patient-reported outcomes. Changes were partially assessed bidirectionally (improvement or worsening) and partially based on EHRA scale. However, EHRA classification was not used for all patients. Consequently, a bidirectional model of symptom change was applied to ensure consistent assessment across the entire study population. While no formalized symptom score was prospectively used in all patients, the assessment strategy reflects real-world clinical practice. These hard endpoints were assessed by outpatient visits and based on the hospital electronic database.

### 2.5. Statistical Analysis

Statistical analysis was performed using SPSS 25 software (IBM SPSS Statistics 25 (IBM Corp., Armonk, NY, USA)). Distribution was verified using a Shapiro–Wilk test. Continuous variables were expressed as the mean ± standard deviation for normally distributed data or as median with interquartile range (25th–75th percentile) for non-normally distributed data. Group comparisons were performed using Student’s *t*-test or Mann–Whitney U test, as appropriate. Categorical variables (reported as absolute numbers with percentages) were tested using the chi-square test. Multivariable logistic regression was performed to analyze the potential predictors of AF recurrence after PVI and MACCE occurrence in the whole cohort. Time-to-event analyses were performed using the Kaplan–Meier method, with comparisons between groups conducted using the log-rank test. *p* values < 0.05 were considered to be statistically significant, while *p* values between 0.05 and 0.1 were interpreted as indicating a statistical trend.

## 3. Results

### 3.1. The Study Group Characteristics

The study cohort comprised 105 participants, including 55 women (51.4%) and 51 men (48.6%), hospitalized in the cardiology department between January 2017 and June 2024. The mean age of the patients at the time of PVI was 65.2 ± 9.5 years. Treatment with SGLT2 inhibitors, administered to 52 participants hospitalized from mid-2023, was used as a grouping factor. The baseline characteristics of the subgroups are presented in Table 1.

Considering the differences between the subgroups, the prevalence of diabetes mellitus (DM) (62.3% vs. 21.2%) and obesity (75.5% vs. 52%) were significantly higher in the SGLT2i subgroup, although median BMI did not differ significantly. Apart from frequency of DM, the prevalence of other comorbidities, such as arterial hypertension, obstructive sleep apnea, hyperlipidemia, and smoking, as well as CHA_2_DS_2_-VA scores, did not differ significantly between the subgroups, indicating a comparable baseline cardiovascular risk (Table 1). Most individuals in both subgroups were classified as EHRA class IIb (2.5 (2.5–3) in both subgroups), suggesting moderate symptom burden related to AF. The baseline EHRA distribution according to the subgroups was as follows: in the SGLT2i group: I-3, IIa-8, IIb-25, III-17, and IV-1; and in control group: I-1, IIa-5, IIb-22, III-21, and IV-3. Data concerning concomitant diseases are presented in Table 2.

Echocardiographic findings did not differ significantly between the subgroups (Table 3).

The treatment considering anticoagulant type, as well as antiarrhythmic drugs prescribed after the ablation is described in Table 4.

### 3.2. Outcomes After a One-Year Observation

Re-evaluation was performed one year after the ablation based on arrhythmia parameters recurrence and symptom reduction, as well as hard endpoints–severe bleeding events, stroke/TIA, cardiovascular events, hospitalization due to cardiovascular reasons, all-cause mortality and, collectively, MACCE. The results of the reassessment are presented in Table 5.

During the one-year follow-up, arrhythmia recurrence occurred in 31.7% of the whole study group, with no significant difference between the groups (24.5% in SGLT2i vs. 39.2% in controls, *p* = 0.108). Clinical improvement in symptoms was reported in 76.9% of patients and occurred significantly more frequently in the SGLT2i group compared to the non-SGLT2i group (90.6% vs. 62.7%, *p* = 0.001). A statistical trend suggested that SGLT2i administration was associated with a lower incidence of all-cause mortality (5.4% vs. 0%; *p* = 0.076). No significant differences were observed between the subgroups in the rates of cardiovascular events, strokes/TIAs, cardiovascular hospitalizations and MACCE. No severe bleeding events occurred in the study sample.

**Figure 2:**
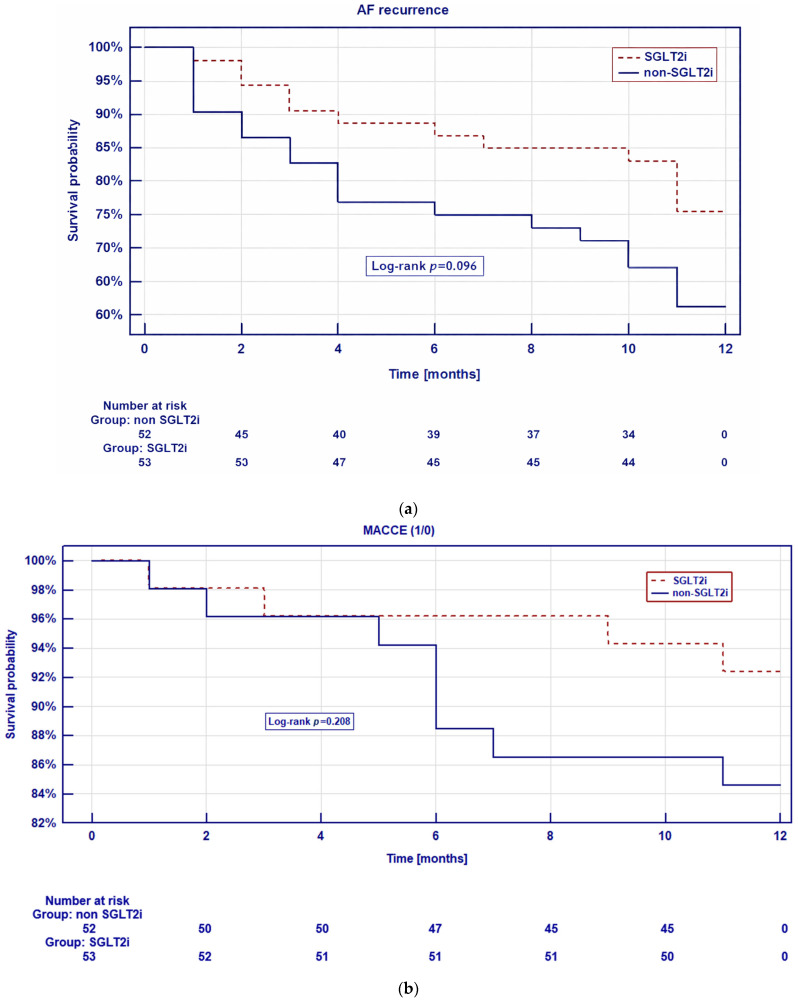
(**a**) Kaplan–Meier curves for event-free survival of AF recurrence comparing patients treated with SGLT2 inhibitors and individuals not receiving SGLT2 inhibitors (non-SGLT2i). The *p*-value was calculated using the log-rank test. (**b**) Kaplan–Meier curves for event-free survival of MACCE occurrence comparing patients treated with SGLT2 inhibitors and individuals not receiving SGLT2 inhibitors (non-SGLT2i). The *p*-value was calculated using the log-rank test.

Kaplan–Meier curves for event-free survival of AF recurrence and MACCE occurrence according to SGLT2i use are presented in Figure 2a and Figure 2b, respectively. The analysis showed a statistical trend for lower risk AF recurrence in the SGLT2i subgroup compared to the controls (log-rank *p* = 0.096). MACCE occurrence risk was similar in both subgroups (log-rank *p* = 0.208).

**Figure 3:**
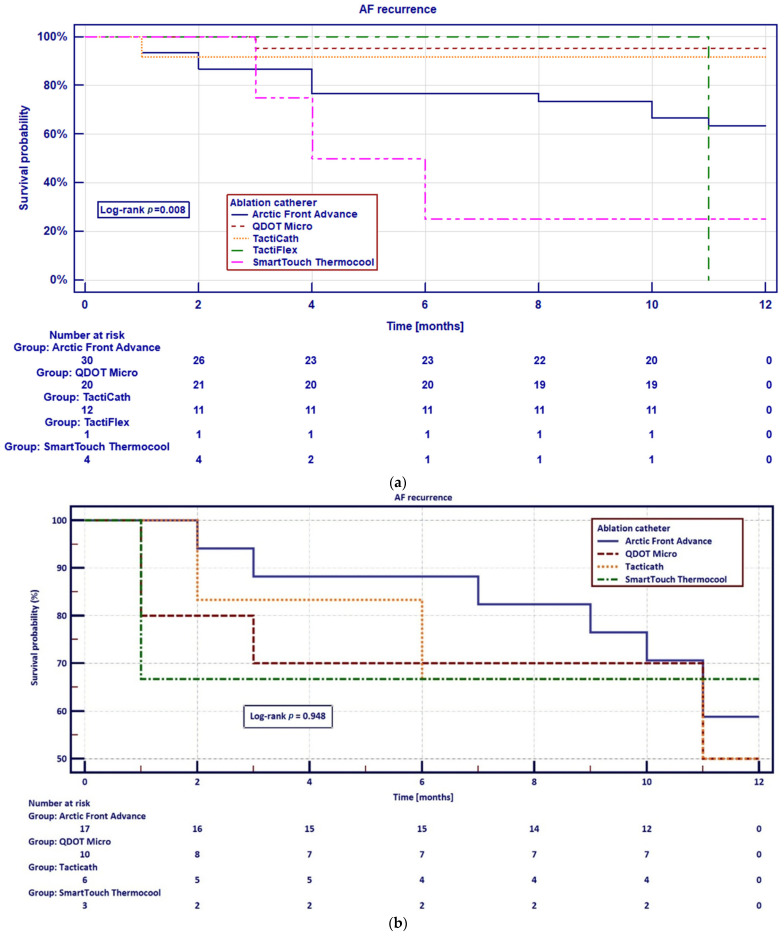
(**a**) Kaplan–Meier curves for event-free survival of AF recurrence in patients with paroxysmal AF, stratified by catheter type. The *p*-value was calculated using the log-rank test. (**b**) Kaplan–Meier curves for event-free survival of AF recurrence in patients with persistent AF, stratified by catheter type. The *p*-value was calculated using the log-rank test.

Kaplan–Meier analysis for event-free survival of AF recurrence was performed and stratified by the catheter, utilized during ablation. In patients with paroxysmal AF there was a significant difference in the primary endpoint across catheter types (log-rank *p* = 0.008), while no significant differences were observed in patients with persistent AF (log-rank *p* = 0.948) (Figure 3a and Figure 3b, respectively).

### 3.3. Multivariable Analysis of Selected Endpoints

The stepwise logistic regression analysis was performed for arrhythmia recurrence after a one-year follow-up period. It demonstrated that NOACs administration was independently associated with a lower risk of AF recurrence (OR 0.27; 95% CI 0.096–0.77; *p* = 0.014). The model had a very good fit reflected by Hosmer–Lemeshow *p*-value of 1.0 and a moderate predictive power reflected by area under curve (AUC) of 0.609, 95% CI: 0.504–0.707.

**Figure 4:**
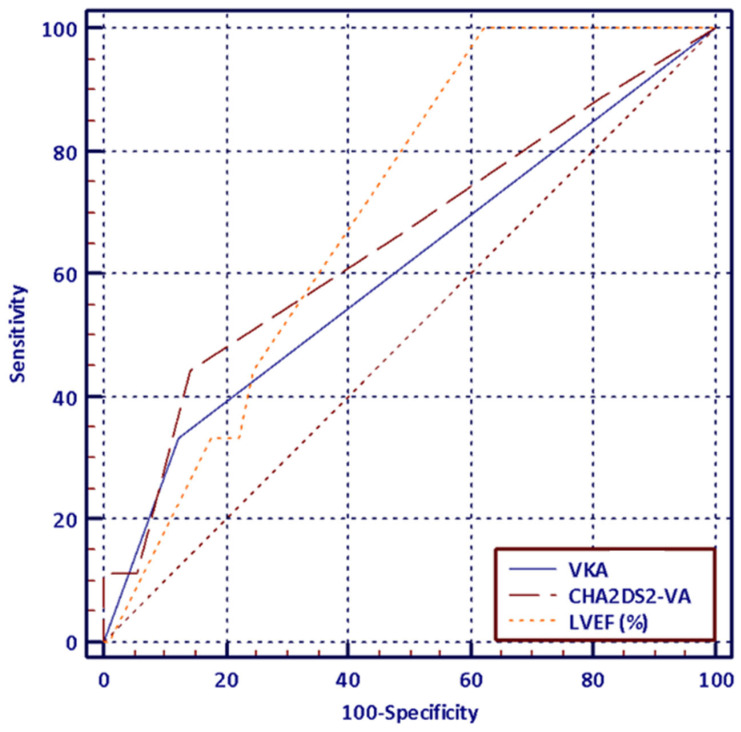
The receiver operating characteristic (ROC) curve analysis for evaluating the predictive accuracy of the CHA_2_DS_2_-VA, LVEF and VKA use in MACCE occurrence in HFpEF population in one-year follow-up after the first-time PVI.

The stepwise logistic regression analysis revealed that MACCE occurrence during a one-year follow-up after the first-time PVI in HFpEF population was predicted by higher CHA_2_DS_2_-VA score (OR 5.63; 95% CI 1.57–20.12; *p* = 0.008), lower LVEF (OR 0.74; 95% CI 0.57–0.99; *p* = 0.028) and VKA use (OR 97.44; 95% CI 3.2–2962.57; *p*= 0.009). The model was characterized by a very good fit reflected by Hosmer–Lemeshow’s *p*-value of 0.99 and a very good predictive power (area under the curve (AUC) 0.90; 95% CI: 0.805–0.959). (Figure 4 and Table 6)

## 4. Discussion

This retrospective study aimed to evaluate the potential impact of SGLT2 inhibitor pharmacotherapy on clinical outcomes and procedural efficacy in patients with HFpEF and AF undergoing the first-time PVI. Our findings suggest that treatment with SGLT2 inhibitors is associated with significant clinical improvement, particularly in symptom relief. Furthermore, a trend toward reduced all-cause mortality was observed. No significant effects on other clinical outcomes, including arrhythmia recurrence, were reported.

It is important to note that our results should be interpreted regarding the time-based cut-off, applied to divide the cohorts, which coincides with the European Society of Cardiology (ESC) guidelines supplementation of SGLT2i use in HFpEF therapy. Therefore, the observed differences may partly reflect changes in clinical practice supported by evolving guideline recommendations rather than representing a purely independent treatment effect. The time-based cohort definition may be influenced by recent years, which have witnessed substantial technological advancements, ranging from the development of novel techniques such as pulsed-field ablation to refinements in procedural equipment, including contact force- sensing catheters and optimized lesion indexing algorithms [20]. On the other hand, 2020 ESC guidelines for AF management emphasized the preferential use of NOACs over VKA in patients undergoing PVI. This strategy has been maintained over time [21].

Mechanistically, SGLT2 inhibitors are hypothesized to exert antiarrhythmic effects through multiple pathways, including reduction in left atrial pressure and wall stress, improved diastolic function, attenuation of myocardial fibrosis and anti-inflammatory effects. Results from animal models highlight the role of improved myocardial mitochondrial function. Reduction in reactive oxygen species (ROS) production, via an adenosine monophosphate-activated protein kinase (AMPK) dependent mechanism, decreases the extent of fibrosis. Experimental studies have shown that SGLT2 inhibition can reduce atrial conduction heterogeneity and fibrosis, potentially stabilizing atrial electrophysiology. Moreover, SGLT2 inhibitors may improve autonomic tone, reduce epicardial adipose tissue, and exert antioxidant effects. These beneficial effects may contribute to reduction in arrhythmogenic substrate and improvement for rhythm control strategies such as PVI [22]. The main antifibrotic mechanism is related to the suppression of fibroblasts activation and proliferation, as well as their transition from the endothelium to the extracellular matrix. This process is dependent on AMPK activity, which ultimately prevents excessive collagen production. In experimental HFpEF models, SGLT2 inhibition was associated with reduced arrhythmogenic spontaneous Ca^2+^ release events and improved cellular Ca^2+^ handling, as well as enhanced activity of the sodium–calcium exchanger (Na^+^/Ca^2+^ exchanger, NCX), which was related to reduced arrhythmogenic potential [23].

A meta-analysis of 75,279 patients compared the effects of SGLT2 inhibitors therapy with placebo or no treatment on AF burden and clinical implications. SGLT2 inhibitors were associated with lower risk of serious AF events defined as life-threatening, death-causing, adverse or prolonged hospitalization, disability, substantially impaired normal physiological or functional capacity, or exposed the patient to a significant risk necessitating medical or surgical intervention to avert any of the previously described outcomes (RR (relative risk) 0.76 [95% CI, 0.64–0.90], I^2^ = 0%, *p* = 0.8). These results were also obtained in specific populations including DM, chronic kidney disease, cardiovascular risk factors and HF with reduced ejection fraction (HFrEF), as well as in follow-up longer and shorter than 2 years. What is more, patients with DM and AF treated with SGLT2 inhibitors were at 30% lower risk of cardiovascular death and HF hospitalization compared to placebo (95% CI, 0.57–0.85) [24].

In previous studies SGLT2 inhibitors have been associated with decreased relative risk of AF and atrial flutter (AFl) cumulatively, in patients with type 2 of DM. A meta-analysis by Li W.J. et al. [25] showed lower rates of HF development in the SGLT2 inhibitors subgroup compared with the subgroup with no such a treatment. However, no correlation between AF/AFl development and HF was proven. These findings are consistent with the evidence demonstrating the favorable hemodynamic and metabolic effects of SGLT2 inhibitors, which extend beyond glycemic control to reduction in left ventricular filling pressure, inhibition of atrial fibrosis, improved vascular compliance and enhanced diuresis without neurohormonal activation. These outcomes also suggest the favorable impact of SGLT2 inhibitors therapy duration on arrhythmic burden [25]. In contrast, more recent meta-analysis by Ouyang X suggests that the SGLT2 inhibitors in HF patients may show limited benefit in reducing arrhythmic burden in comparison to its effects in broader clinical populations. The effect of dapagliflozin and empagliflozin on reducing AF incidence was assessed considering the follow-up duration and heart failure type. Neither the specific SGLT2 inhibitor used (dapagliflozin or empagliflozin), the duration of follow-up (above or below one year), nor the type of heart failure (HFpEF or HFrEF) demonstrated superiority over placebo in reducing AF prevalence [26].

The effect of SGLT2 inhibitors was evaluated in relation to post-PVI outcomes in the type 2 of DM population. This pharmacotherapy was associated with a lower cumulative incidence of the necessity of the intervention defined as electrical cardioversion, new initiation of class I or III AAD and repeat PVI. These results indicate a reduction in arrhythmia recurrence rates in this subgroup. Despite the fact that we did not achieve significant differences in this endpoint, the arrhythmia recurrence rate was numerically lower than in the non-SGLT2i subgroup (24.5% vs. 39.2%; *p* = 0.108) [27].

The overall arrhythmia recurrence rate was 31.7% in our study at a one-year observation period, while previous studies indicate higher incidences of it in the HFpEF population, but the follow-up duration was usually longer [28]. Moreover, HFpEF patients exhibited the greatest rate of arrhythmia recurrence among heart failure patients stratified by ejection fraction [29,30].

The recent analysis based on the China-AF Registry by Zhao Z. et al. [31] demonstrated significant reduction in AF recurrence rates in the SGLT2i subgroup, regardless of the type of heart failure as defined by LVEF. Additionally, the combined incidence of cardiovascular death, thrombotic events, and cardiovascular hospitalization was also lower in this subgroup. However, in contrast to our findings, a reduction in all-cause mortality was not correlated with SGLT2 inhibitors treatment. Moreover, similar to our study, differences in baseline characteristics were observed in primary outcomes. Due to the limited sample size, propensity score matching could not be performed in our analysis [31]. Another relevant effect of SGLT inhibitors is their impact on arrhythmias—atrial, as well as ventricular. Shih-Jie Jhuo et al. report that the T2DM population with controlled arterial hypertension treated with SGLT2 inhibitors presented lower incidence of cardiac arrhythmias in general, with HR of 0.58 (*p* = 0.013) [32]. SGLT2 inhibitors showed reduced risk of ventricular arrhythmias compared to the patients treated with inhibitors of dipeptidyl peptidase 4 (DPP-4) [33]. It may indicate that the lower incidence of all-cause mortality observed in the group receiving SGLT2 inhibitors could be also linked to a possible effect of lowering the incidence of ventricular arrhythmias.

While our study did not identify a significant reduction in arrhythmia recurrence after PVI in the SGLT2i subgroup, the recurrence rate was numerically lower, and the absence of any deaths in this group compared to three in the control group is notable. Similarly, the incidence of MACCE and cardiovascular hospitalizations was numerically reduced in the SGLT2i subgroup. Although these findings did not reach statistical significance, likely due to sample size limitations, they are consistent with existing data from major trials mentioned before. The advantage of our study is the selected study group—the patients with HFpEF and AF undergoing the first-time PVI—a population often underrepresented in clinical trials.

Data so far, obtained from meta-analysis by Anand N Ganesan, revealed increasing efficacy of multiple PVI with the 1-year success rate of 85.7% (95% CI 81.9% to 88.7%) compared to the single procedure 1-year success rate in these studies, which achieved 65.3% (95% CI 57.5–72.4%) [34]. Although in a more recent study multiple ablations did not demonstrate superiority over a single procedure in terms of arrhythmia recurrence rates (43.8% vs. 41.9%, *p*  =  0.37), mortality (0.3% vs. 0.1%, *p*  =  0.39) and MACCE (0.8% vs. 0.6%, *p*  =  0.47) during one-year follow-up, there is still a limited number of studies on this topic [35]. In this context our study gives a valuable contribution to the ongoing research discussion.

The stepwise logistic regression in our study identified that NOACs administration was independently associated with a lower risk of AF recurrence. Contrary to our result, a study by Wen S. et al. indicated use of NOACs as an independent predictor of arrhythmia recurrence among the subgroup with persistent AF (HR 1.39, 95% CI 1.07–1.81, *p* = 0.013), but not in the whole study sample [36]. The beneficial association between NOAC use and AF recurrence after ablation observed in our study may be related to the pleiotropic effects of activated factor X inhibition on atrial remodeling. Experimental data demonstrate that factor Xa stimulates atrial endothelial and interstitial cells, promoting oxidative stress, inflammation, and fibrosis, whereas its inhibition counteracts these profibrotic responses [37]. Similar effects were observed in animal models of congestive heart failure. Edoxaban therapy suppressed atrial fibrosis and reduced expression of profibrotic mediators (protease-activated receptor-2 (PAR-2) and fibronectin), suggesting that NOACs may directly modulate the structural substrate of atrial arrhythmogenesis [38].

To our knowledge, data considering predictors of AF recurrence after PVI in HFpEF patients is limited. However, factors associated with recurrence in general population are well established and remain: non-paroxysmal AF, estimated glomerular filtration rate (eGFR), bundle branch block, heart failure, arterial hypertension, obesity, left atrial volume index (LAVi), elevated inflammatory biomarkers [39,40,41].

Our analysis showed that VKA use, higher CHA_2_DS_2_-VA score and lower LVEF (in the population with preserved LVEF) were independent predictors of MACCE occurrence in the study group. Higher CHA_2_DS_2_-VA was also described as an independent predictor of adverse cardiac events (cardiac death, acute coronary syndrome, stroke/TIA), AF recurrence and all-cause rehospitalization [42]. These results emphasize the influence of cardiovascular risk burden on fibrosis and adverse myocardial remodeling, as reported in previous studies, which was also reflected in cardiac magnetic resonance imaging by left ventricular late gadolinium enhancement (LV-LGE) [43]. Although the predictive value of higher CHA_2_DS_2_-VA score was previously described, the advantage of our study is the fact that, to our knowledge, MACCE predictors, specifically HFpEF and AF after the first-time PVI, one-year postprocedural population were never assessed.

Future prospective studies with larger cohorts are warranted to validate these findings. Moreover, randomized trials incorporating advanced imaging techniques such as cardiac MRI may better elucidate the impact of SGLT2 inhibitors on atrial structural remodeling and fibrosis, which are key determinants of ablation outcomes.

### Limitations

Our findings should be interpreted considering the study’s limitations. First of all, the retrospective design is subject to confounding and selection bias. Though baseline characteristics were generally homogenic, the differences in comorbidities burden may have influenced treatment decisions and outcomes. The study population comprised all patients who met the inclusion criteria during the observation period. We aimed to minimize variability in procedure techniques and treatment indications. Unfortunately, this resulted in heterogeneity in the baseline characteristics, including factors that may influence the study endpoints. Secondly, the relatively small sample size limits the statistical power to detect differences in clinical events, particularly those with low incidence such as mortality or stroke. Consequently, also the use of propensity score matching was precluded. What is more, the data on the exact time from the first AF episode to PVI was difficult to assess on the basis of the hospital database and this effect was not evaluated. Although EHRA classification was not used in all patients, which precluded application of a standardized symptom score, the bidirectional assessment allowed for consistent evaluation across the study population. In addition, the one-year observation period might be insufficient for a comprehensive assessment of the clinical course, patient characteristics or endpoints. We do not have exact information on the time of arrhythmia recurrence, so we calculated only logistic regression. Notably, the small number of outcome events allowed a limited number of predictors in the logistic regression models. We used stepwise regression, and while the models provide insight into independent predictors, results should be interpreted cautiously.

Despite the limitations, this study provides valuable insight into the interaction between SGLT2 inhibitor therapy and rhythm control strategies in a highly relevant clinical population.

## 5. Conclusions

In conclusion, while our study did not demonstrate a significant reduction in arrhythmia recurrence rates associated with SGLT2 inhibitor use, the observed trends toward lower mortality and improvement in clinical symptom burden highlight the value of SGLT2 inhibitors in the integrated management of patients with HFpEF and AF undergoing the first-time PVI. Additionally, although overall AF recurrence rates were similar between subgroups, Kaplan–Meier analysis showed a non-significant trend toward lower recurrence in the SGLT2i group. However, these results should be interpreted in the context of study’s limitations and heterogeneity of the study population considering comorbidities and treatment, which are proven to influence the endpoints. Our findings contribute to the increasing development and evidence of support for the pleiotropic cardiovascular benefits of SGLT2 inhibitors and suggest a probable beneficial role in rhythm control strategies beyond pharmacologic HFpEF management. The findings should, therefore, be interpreted as hypothesis-generating rather than confirmatory. To sum up, while this study suggests a potential association that adds weight to the ongoing discourse on the pleiotropic and possibly beneficial effects of SGLT2 inhibitors, further investigations are warranted to substantiate these observations.

## Figures and Tables

**Table 1:** Baseline characteristics of the study group.

Variable	Non-SGLT2i Subgroup (*n* = 52) *n* (%) or Mean ± SD or Median (1–3 Quartile)	SGLT2i Subgroup (*n* = 53) *n* (%) or Mean ± SD or Median (1–3 Quartile)	*p* Value
Male, *n* (%)	25 (48.1)	26 (49.1)	0.92
Age, years	65.0 ± 9.9	65.5 ± 9.2	0.758
BMI, kg/m^2^	31.5 ± 4.1	32.7 ± 5.0	0.227
EHRA	2.5 (2.5–3)	2.5 (2.5–3)	0.154
CHA_2_DS_2_-VA	2.4 ± 1.2	2.8 ± 1.1	0.068
NYHA	2.0 (2.0–2.0)	2.0 (1.0–2.0)	0.025
Cryoablation, *n* (%)	27 (51.9)	21 (39.6)	0.206
RF ablation, *n* (%)	25 (48.1)	32 (60.4)	0.206
Paroxysmal AF, *n* (%)	37 (71.1)	31 (58.5)	0.174
Non-paroxysmal AF, *n* (%)	15 (28.9)	22 (41.5)	0.265

AF—Atrial Fibrillation; BMI—Body Mass Index; CHA_2_DS_2_-VA—Congestive Heart Failure, Hypertension, Age ≥ 75 years, Diabetes Mellitus, Stroke/Transient Ischemic Attack/Thromboembolism, Vascular Disease, Age 65–74 years; EHRA—European Heart Rhythm Association; *n*—Number; NYHA—New York Heart Association; RF—Radiofrequency; SD—Standard Deviation; SGLT2i—Sodium-Glucose Cotransporter 2 Inhibitors.

**Table 2:** Prevalence of comorbidities according to the subgroups.

Variable	Non-SGLT2i Subgroup (*n* = 52) *n* (%) or Mean ± SD or Median (1–3 Quartile)	SGLT2i Subgroup (*n* = 53) *n* (%) or Mean ± SD or Median (1–3 Quartile)	*p* Value
Arterial hypertension, *n* (%)	50 (96.2)	48 (90.6)	0.251
DM, *n* (%)	11 (21.2)	33 (62.3)	<0.001
Hyperlipidaemia, *n* (%)	36 (69.2)	42 (80.8)	0.174
Obesity, *n* (%)	26 (52.0)	40 (75.5)	0.013
Sleep apnea, *n* (%)	3 (5.8)	6 (11.3)	0.31
Nicotinism, *n* (%)	3 (5.8)	4 (7.5)	0.715
Ischemic heart disease, *n* (%)	11 (21.2)	12 (22.6)	0.854
Ischemic stroke/TIA, *n* (%)	3 (5.8)	4 (7.5)	0.715

DM—Diabetes Mellitus; *n*—Number; SD—Standard Deviation; SGLT2i—Sodium-Glucose Cotransporter 2 Inhibitors; TIA—Transient Ischemic Attack.

**Table 3:** Baseline echocardiography parameters.

Variable	Non-SGLT2i Subgroup (*n* = 52) *n* (%) or Mean ± SD or Median (1–3 Quartile)	SGLT2i Subgroup (*n* = 53) *n* (%) or Mean ± SD or Median (1–3 Quartile)	*p* Value
LVEF (%)	56.7 ± 4.3	55.0 ± 4.7	0.133
LA (mm)	42.4 ± 4.4	41.5 ± 5.8	0.249
LA area (mm^2^)	24.6 ± 5.2	24.1 ± 4.9	0.808
LVEDD (mm)	50.8 ± 6.2	50.2 ± 6.0	0.674
LVESD (mm)	31.6 ± 6.0	31.2 ± 6.2	0.583
IVS (mm)	11.4 ± 1.9	11.3 ± 1.5	0.881
PW (mm)	9.7 ± 1.4	10.1 ± 1.3	0.119
LVM (g)	198.8 ± 63.9	199.8 ± 56.9	0.792
LVMi (g/m^2^)	94.5 ± 29.5	98.0 ± 24.1	0.655
RWT (m/s)	0.4 ± 0.1	0.4 ± 0.1	0.108
E wave (m/s)	0.9 ± 0.2	0.8 ± 0.3	0.373
A wave (m/s)	0.8 ± 0.2	0.8 ± 0.3	0.238
E/A	1.2 ± 0.6	1.3 ± 0.7	0.68

A—Late Diastolic Transmitral Flow Velocity; E—Early Diastolic Transmitral Flow Velocity; IVS—Interventricular Septum; LA—Left Atrium; LVEDD—Left Ventricular End-Diastolic Diameter; LVEF—Left Ventricular Ejection Fraction; LVESD—Left Ventricular End-Systolic Diameter; LVM—Left Ventricular Mass; LVMi—Left Ventricular Mass Index; *n*—Number; PW—Posterior Wall; RWT—Relative Wall Thickness; SD—Standard Deviation; SGLT2i—Sodium-Glucose Cotransporter 2 Inhibitors.

**Table 4:** The treatment data.

Variable	Non-SGLT2i Subgroup (*n* = 52) *n* (%) or Mean ± SD or Median (1–3 Quartile)	SGLT2i Subgroup (*n* = 53) *n* (%) or Mean ± SD or Median (1–3 Quartile)	*p* Value
NOAC intake, *n* (%)	46 (92)	35 (66)	0.02
VKA intake, *n* (%)	2 (4)	12 (24.5)	0.003
AAD intake before PVI, *n* (%)	30 (62.5)	34 (68)	0.567
Amiodarone intake after PVI, *n* (%)	25 (48.1)	23 (43.4)	0.63
Sotalol intake after PVI, *n* (%)	6 (11.5)	2 (3.8)	0.134
Propafenone intake after PVI, *n* (%)	11 (21.2)	12 (22.6)	0.854
Flecainide intake after PVI, *n* (%)	0 (0)	0 (0)	-

AAD—Antiarrhythmic Drug; *n*—Number; NOAC—Non-Vitamin K Antagonist Oral Anticoagulant; PVI—Pulmonary Vein Isolation; SD—Standard Deviation; SGLT2i—Sodium-Glucose Cotransporter 2 Inhibitors; VKA—Vitamin K Antagonist.

**Table 5:** The outcomes regarding hard endpoints after a one-year follow-up.

Variable	Non-SGLT2i Subgroup *n* (%)	SGLT2i Subgroup *n* (%)	*p* Value
Arrhythmia recurrence, *n* (%)	20 (39.2)	13 (24.5)	0.108
Symptom reduction, *n* (%)	32 (62.7)	48 (90.6)	0.001
All-cause mortality, *n* (%)	3 (5.8)	0 (0)	0.076
Hospitalization due cardiovascular reason, *n* (%)	4 (7.7)	4 (7.5)	0.978
Cardiovascular events, *n* (%)	0 (0)	1 (1.9)	0.32
Stroke/TIA, *n* (%)	1 (1.9)	0 (0)	0.31
Severe bleeding events, *n* (%)	0 (0)	0 (0)	-
MACCE, *n* (%)	6 (11.5)	4 (7.5)	0.486

MACCE—Major Adverse Cardiac and Cerebrovascular Events; *n*—Number; TIA—Transient Ischemic Attack.

**Table 6:** The details of the analysis evaluating the predictive accuracy of the CHA_2_DS_2_-VA, LVEF and VKA use in MACCE occurrence in HFpEF population in one-year follow-up after the first-time PVI.

Area under the ROC curve (AUC)	0.9
Standard error	0.05
95% Confidence interval	0.81–0.96

## Data Availability

The data presented in this study are available on request from the corresponding author due to (specify the reason for the restriction).

## Abbreviations

The following abbreviations are used in this manuscript:

HFpEF	heart failure with preserved ejection fraction.
HFrEF	heart failure with reduced ejection fraction.
AF	atrial fibrillation.
SGLT2i	sodium-glucose cotransporter inhibitors.
DM	diabetes mellitus.
LVEF	left ventricle ejection fraction.
LA	left atrium.
LAVi	LA volume index.
IVS	interventricular septum.
PW	posterior wall.
LVM	left ventricular mass.
LVMi	LVM index.
RWT	relative wall thickness.
LVESV	LV end-systolic.
LVEDV	LV end-diastolic volumes.
eGFR	estimated glomerular filtration rate.
NT-proBNP	N-terminal pro-B-type natriuretic peptide.
PVI	pulmonary veins isolation.
AAD	antiarrhythmic drugs.
TIA	transient ischemic attack.
MACCE	major adverse cardiovascular and cerebrovascular events.
ROC	receiver operating characteristic.
HR	hazard ratio.
ROS	reactive oxygen species.
AMPK	activated protein kinase.
NCX	Na^+^/Ca^2+^ exchanger.
DPP-4	dipeptidyl peptidase 4.
PAR-2	protease-activated receptor-2.
